# Pure Water

**Published:** 1882-07

**Authors:** 


### PURE WATER.

There is nothing so essential to health and comfort as an abundant supply of pure, fresh water ; we partake of it more abundantly than we do of any other substance, since, in addition to that taken in various forms to allay thirst, whatever enters the stomach as food is largely saturated with it; indeed, four-fifths of the weight of our bodies is water. If the source of supply is contaminated by decayed vegetable matter, by cess-pools and sewage from defective drainage, which is more frequently the case than is generally believed, such water is certain sooner or later to induce disease.

Nature seems, in some unaccountable manner, to tolerate abuses for a long time ; hence it is one of the most difficult undertakings in the world to convince the average man that all well water is not pure and wholesome, even though his well may be filled to overflowing, after a heavy rain, with the washings from a barn yard. There are but few open wells throughout the country where the depth of water is not increased in proportion to the amount of rain-fall. Nearly all this increase consists of surface water that is impregnated with whatever the soil contains for a considerable distance around ; not to mention the drowned insects and worms that are frequently carried along in quantities sufficient to render the water putrid with their decaying bodies. I believe that this poisonous water supply, is the chief source of all forms of malarial fever, and that that *bête noir* called *miasma*, which is supposed to emanate from the decaying vegetation of marshy ground, but whose form and substance all the appliances of modern science have failed to detect, may have less to do with these forms of disease, than is generally supposed. I know of families that have resided near marsh lands, in a so-called "fever and ague district" for many years, that have never had fever and ague, while others supposed to be more favorably located have suffered greatly from the various types of this malady.

I have in several instances inquired into the causes of the immunity from sickness in one case, and it its causes in the other, and the views here stated have been invariably confirmed.

I know of a house, located near a marsh, and where half the families in its vicinity suffer from malarial fevers.

Two families had occupied the house at different times, and both left on account of sickness. All the water they used was supplied from an open well, 10 feet deep, in the lowest portion of the door yard. A third family purchased the property for less than half its cost, made a " driven well" 25 feet deep, from which they get all the water used for drinking and cooking. They have occupied the premises over four years, and enjoy almost perfect health.

### HOW TO GET PURE WATER.

In some sections of the country it is not possible to get good water except at great expense. In that case, rain water may be saved in cement cisterns or iron tanks, and kept pure for a long time with proper care ; but, where it is practicable, no water supply is so safe as that procured from " driven wells." But, after thousands of "driven wells" were constructed, a patent was granted—unexpectedly and as some think unjustly—the vital claim of which is for " a well, to the lining of which a pump is attached." In plain words, for a well made by driving down an iron pipe to the water, and screwing on a pump. For this privilege the patentee claims $10. Fortunately, there is an easy road around this obstruction ; and a far better well, in some respects, can be made outside this patent. If the soil is tolerably free from stones, a well can usually be made for a few dollars on the following plan : Get enough galvanized iron pipe, of one and a quarter-inch caliber, to reach the water. Have it cut in lengths convenient to drive—say 6 to 8 feet—and couplings to screw the pieces together. Then get a piece of plain iron tube, same size and 2 feet long. Have about 100 quarter-inch holes drilled through this piece of pipe, to admit the water and sand. Then have a blacksmith weld a piece of iron 6 inches long, and size of caliber, into one end of this short piece, leaving about 3 inches out, which he must hammer down to a point, not sharp, but blunt and strong; have him harden it so that it will split and penetrate quite large stones on its passage down. Screw on one length of pipe, and then, placing the point where you want the well, have one man hold a wooden beetle on the upper end of the pipe, while another man strikes the beetle with a heavy sledge hammer It will take from two hours to two days to drive down twenty feet of pipe, depending on the nature of the soil. With a small piece of iron or lead attached to a string, soundings for water can be made from time to time as the work proceeds. It is best to drive some two or three feet after water is reached, but not to go entirely through it. When the pipe is thus driven down, it is really an open well, it matters not if it be 1 inch in diameter or 6 feet, it is an open well nevertheless, and no man can contest your right to make such a well. Having now a well, get a three-quarter-inch galvanized pipe, attach a pump to it, and put it in the well. Pump out the sand, and you have a well into which impurities can not enter. By making the well of 1½ inch pipe, an inch pipe could be used with the pump; but I believe the well described of sufficient size to supply water for fifty families. In places where a "driven well" is impracticable, or where it is desirable to utilize an open well, either of the following plans may be adopted. Clean out the well, then get a galvanized iron pipe 1 inch in diameter and long enough to reach to within a foot of the bottom of well, and attach a pump to it. Plug up lower end of pipe with a piece of iron or hard wood. Drill 20 or 30 quarter-inch holes through the pipe near the lower end, to admit the water. Put a large stone in the center at bottom of well, on which to stand the pipe. Pack large round or "cobble" stones around the pipe, up 3 or 4 feet; then smaller stones, and next gravel and sand. Then pack in clay until the well is filled. This becomes practically a "driven well." The second plan is, after cleaning out the well, to line it with glazed earthen pipes. Put the pieces one on the other, packing outside with clay, and putting cement around the joints until the top is reached. This will exclude the surface water. Pipes of any diameter may be used—from 6 inches to 2 feet. If people would heed these suggestions, much sickness and suffering might be prevented.

---

Putting Coins in the Mouth.—We often see persons put a piece of money between the lips to hold it while their hands are occupied in putting on their gloves or adjusting the dress. It is not only untidy, but it is unsafe, because coins may thus convey the seeds of disease.

